# Improved clinical outcome after invasive management of patients with recent myocardial infarction and proven myocardial viability: primary results of a randomized controlled trial (VIAMI-trial)

**DOI:** 10.1186/1745-6215-13-1

**Published:** 2012-01-03

**Authors:** Ramon B van Loon, Gerrit Veen, Leo HB Baur, Otto Kamp, Jean GF Bronzwaer, Jos WR Twisk, Freek WA Verheugt, Albert C van Rossum

**Affiliations:** 1 Department of Cardiology, VU University Medical Center, Amsterdam, The Netherlands; 2 Department of Cardiology, Atrium Medical Center Parkstad, Heerlen and Faculty of Health, Medicine and Life Sciences, University Maastricht, The Netherlands; 3 Department of Clinical Epidemiology and Biostatistics, VU University Medical Center, Amsterdam, The Netherlands; 4 Heartcenter, University Medical Center, St Radboud, Nijmegen, The Netherlands

## Abstract

**Background:**

Patients with ST-elevation myocardial infarction (STEMI) not treated with primary or rescue percutaneous coronary intervention (PCI) are at risk for recurrent ischemia, especially when viability in the infarct-area is present. Therefore, an invasive strategy with PCI of the infarct-related coronary artery in patients with viability would reduce the occurrence of a composite end point of death, reinfarction, or unstable angina (UA).

**Methods:**

Patients admitted with an (sub)acute myocardial infarction, who were not treated by primary or rescue PCI, and who were stable during the first 48 hours after the acute event, were screened for the study. Eventually, we randomly assigned 216 patients with viability (demonstrated with low-dose dobutamine echocardiography) to an invasive or a conservative strategy. In the invasive strategy stenting of the infarct-related coronary artery was intended with abciximab as adjunct treatment. Seventy-five (75) patients without viability served as registry group. The primary endpoint was the composite of death from any cause, recurrent myocardial infarction (MI) and unstable angina at one year. As secondary endpoint the need for (repeat) revascularization procedures and anginal status were recorded.

**Results:**

The primary combined endpoint of death, recurrent MI and unstable angina was 7.5% (8/106) in the invasive group and 17.3% (19/110) in the conservative group (Hazard ratio 0.42; 95% confidence interval [CI] 0.18-0.96; p = 0.032). During follow up revascularization-procedures were performed in 6.6% (7/106) in the invasive group and 31.8% (35/110) in the conservative group (Hazard ratio 0.18; 95% CI 0.13-0.43; p < 0.0001). A low rate of recurrent ischemia was found in the non-viable group (5.4%) in comparison to the viable-conservative group (14.5%). (Hazard-ratio 0.35; 95% CI 0.17-1.00; p = 0.051).

**Conclusion:**

We demonstrated that after acute MI (treated with thrombolysis or without reperfusion therapy) patients with viability in the infarct-area benefit from a strategy of early in-hospital stenting of the infarct-related coronary artery. This treatment results in a long-term uneventful clinical course. The study confirmed the low risk of recurrent ischemia in patients without viability.

**Trial registration:**

ClinicalTrials.gov:  NCT00149591.

## Background

Management of acute myocardial infarction (AMI) has changed considerably over the last two decades [1-3]. Optimal treatment for patients who have acute myocardial infarction with ST-segment elevation includes early reperfusion with primary PCI or thrombolytic therapy. Due to the low availability of primary PCI, most patients with STEMI are treated with intravenous thrombolysis. Even in developed countries many patients receive thrombolytic therapy. Approximately one third of eligible patients do not receive early reperfusion therapy, in many cases because of late presentation [4].

After successful thrombolysis, more than 50% of patients have a significant residual stenosis and about 20-30% suffer from recurrent ischemic events because of plaque-instability in the infarct-related coronary artery [5]. Thus, after initial salvage of myocardium - being the primary goal of thrombolytic therapy - the rescued, viable myocardium is at risk for recurrent ischemia and necrosis. Several studies have indeed shown that after thrombolysis patients with residual viability in the infarct-area are at increased risk of recurrent ischemia or reinfarction [6-13]. Viability in the infarct zone is thought to be a potential substrate for future cardiac events. The impact of revascularization on clinical outcome in patients with viability after AMI was studied in a meta-analysis of non-randomized data [14]. Patients with viable tissue in the infarct area experienced significant less cardiac events after a revascularization procedure.

In contrast, the cardiac event-rate in patients without viability was low and did not change by an invasive strategy. In current clinical practice, viability-testing is not used as a tool for post-myocardial infarction risk-assessment and patient management. We report the results of the Viability-guided Angioplasty after acute Myocardial Infarction (VIAMI) trial, which tested the hypothesis that a strategy of viability guided angioplasty with stenting after AMI in patients treated with thrombolysis or who were too late for reperfusion therapy and remained stable for 48 hours, would reduce the occurrence of a composite end point of death, reinfarction, or unstable angina.

## Methods

### Study population and study design

The methods used in the trial have been described previously [15]. In brief, the VIAMI-trial was a prospective, multicenter, randomized, controlled clinical trial (RCT). Between April 2001 and January 2006, 291 patients were enrolled from 11 participating Dutch hospitals. Patients admitted to any of the participating centers with an (sub)acute myocardial infarction, who were not treated by primary or rescue angioplasty, and who were stable during the first 48 hours after the acute event, were screened for the study.

Stable patients revealed no signs of ongoing ischemia based on electrocardiographic characteristics or persistent chest discomfort. Patients who were admitted within 6 hours after symptom onset, received thrombolysis combined with heparin. Patients admitted more than 6 hours after symptom onset, received only heparin or low weight molecular heparin (LWMH).

Patients < 80 years of age were considered suitable for the study when they met the criteria for definite myocardial infarction, i.e. an significant rise in creatine kinase-MB levels (twice the upper limit of normal: ULN), 1 mm ST segment elevation in two or more standard leads or 2 mm ST segment elevation in two contiguous chest leads, and/or the development of Q waves.

Patients underwent low dose dobutamine echocardiography (LDDE) for the detection of viability within 72 hours after AMI. It is a safe and well-validated bedside test with a diagnostic accuracy of about 80%, which is comparable to scintigraphical techniques (SPECT/PET) [16]. Before the administration of dobutamine, a baseline echocardiogram is performed. Five standard views are obtained: the parasternal long-axis and short-axis view and the apical two, three- and four-chamber view. A 16-segment model is used in which the apex is divided in 4 segments. Segmental wall motion and thickening is scored according to a 4-point scale: 1 = normal, 2 = hypokinetic, 3 = akinetic, and 4 = dyskinetic. Left ventricular volumes and ejection fraction are measured by use of the modified Simpson's rule algorithm from orthogonal apical long-axis projections. Dobutamine is administrated intravenously at doses of 5, 10, and 15 μg/kg/min, for 5 minutes at each dose. When a 10% increase in heart rate is not achieved with 15 μg/kg/min, a 5-minute infusion with 20 μg/kg/min can be used as the final stage of the procedure. This test was performed according to the guidelines of the American Society of Echocardiography [17].

Viability was defined as the improvement of wall motion abnormalities (WMA's) in two or more segments of the infarct zone. Patients without WMA's were not included in this trial. In case of poor acoustic window ultrasound contrast agents were used to improve image quality and diagnostic yield.

All images were sent to the core-lab (VU University Medical Center, Amsterdam, The Netherlands) and were analyzed by 2 experienced observers. A third observer was used in case of disagreement to reach consensus. All eligible patients provided written informed consent. The study complied with the Declaration of Helsinki and all ethics committees of the participating centers approved the protocol.

### Randomization and treatment

Patients with viability in the infarct-area were randomized to an invasive or a conservative treatment strategy. Permuted block randomization was performed with a block size of ten. All patients were treated with aspirin, beta blockers, angiotensin-converting-enzyme inhibitors, statins as accepted by international guidelines [1,2].

The invasive strategy patients underwent in-hospital coronary angiography with the intention to perform PCI with stenting of the infarct-related coronary artery (IRA). In the conservative group an ischemia-guided approach was adopted with stress testing before hospital discharge. After a positive test for ischemia coronary angiography was strongly recommended. If an intervention was performed, it was considered planned and not interpreted as an event. Patients without viability served as a registry group also with a long-term follow-up.

Angiography and PCI was performed as soon as possible after randomization. When a significant (≥ 50%) stenosis or occlusion of the infarct-related coronary artery was found, PCI with stenting was performed when feasible. In all cases where PCI was performed, abciximab was used according to the EPILOG protocol [18]. A bolus of 0.25 mg per kilogram of body weight was administered 10 to 60 minutes before balloon inflation, followed by an infusion of 0.125 μg per kilogram per minute (maximum, 10 μg per minute) for 12 hours. After stenting, all patients received oral clopidogrel with a 300 mg loading dose. In case of severe 3-vessel disease or significant left main stem stenosis, where PCI is judged to be a high risk, coronary artery bypass grafting was to be considered.

### End points

The primary endpoint was the composite of death from any cause, recurrent infarction and unstable angina at 1-year follow-up. Secondary endpoints were the need for revascularization and the occurrence of angina pectoris (Canadian Cardiovascular Society classification (CCS)).

A recurrent myocardial infarction was diagnosed if there was an increase in the total creatine kinase and MB isoenzyme activity (2 times ULN) and either a history of ischemic chest discomfort or electrocardiographic changes indicative for trans mural ischemia or necrosis. Reinfarction during hospitalization required a decrease of cardiac enzymes, followed by a subsequent rise to a level of 2 times ULN and 50% above a previous measured value.

For the diagnosis of unstable angina, patients had to be rehospitalized with ischemic chest pain or discomfort occurring at rest or with minimal exertion. In addition, the need for intravenous medical intervention and/or objective evidence of myocardial ischemia was required.

Members of an independent clinical event and end point committee, who were unaware of the treatment assignments of the patients, adjudicated all end points.

### Statistical analysis

The VIAMI-trial was conducted to investigate the differences in clinical outcome between an invasive and a conservative strategy in patients with demonstrated viability in the infarct-area. The expected event rate in the viability positive group was estimated to be 35 percent. To demonstrate with a power of 80% (α = 0.05, two-sided) that PCI leads to a 50% reduction in event rate in the invasive group compared to the conservative group, 200 patients would be needed in each group.

As a formal stopping rule for the study the following was used: if one of the treatment strategies appeared significantly superior at interim analysis (p ≤ 0.01), the study would be stopped. Interim analysis was performed each time another 100 patients were included.

Baseline descriptive data are presented as a mean ± standard deviations (SD). Differences in clinical and echocardiographic variables are assessed by unpaired Student's t-test. Differences between proportions are assessed by chi-square analysis; a Fisher's exact test is used when appropriate. Event-free survival curves are computed with the Kaplan-Meier method, and the differences between these curves are tested with a log-rank test. The Cox proportional hazards regression analysis was used to estimate the treatment effect as hazard ratio (HR) with 95% confidence intervals. Besides the "crude" effects, adjustments were made for DM, hypertension, hypercholesterolemia, current smoking, family history of CAD (model a), clinical history (angina, myocardial infarction, PCI or CABG) and medication use at baseline (aspirin, beta-blocker, Ca-inhibitor, statins, ACE-I and AT II antagonist) (model b) and for all covariates (model c).

All analyses were performed on an intention-to-treat basis. Outcome per-protocol was also evaluated, since this would reflect the true influence of PCI on clinical outcome. Because after randomization there was a median waiting-time of two days before a revascularization procedure was performed inevitably some events occurred. In the per-protocol analysis these events are excluded from analysis, because they occurred before the by protocol demanded intervention. To make a fair comparison between the two groups in the per-protocol analysis we also excluded the events in the conservative group occurring during the first two days after randomization. All analyses were performed with the use of SPSS software, version 16.0 (SPSS, Inc., Chigago, Illinois).

## Results

### Baseline characteristics

Between April 2001 and January 2006, 216 patients were enrolled in the trial. Of these, 106 patients were randomly assigned to the invasive strategy and 110 patients to the conservative strategy. The pre-specified number of patients was not achieved. This was mainly caused by a slowing down of inclusion-rate in the second half of the study period due to the fast introduction of widely available primary PCI in the Netherlands (> 90%). At the start of this study, 50% of all AMI were still being treated with thrombolysis in the Netherlands.

The baseline characteristics of the patients in the two randomized groups were similar except for a lower prevalence of statin use in the group assigned to the conservative strategy (p = 0.02)(Table 1). The mean age was 60 years with about 80% male patients. Only 5% experienced a prior myocardial infarction and about 3% a revascularization procedure in the past. Almost half of the study population was actively smoking. Time from onset of symptoms, caused by the index infarction, to randomization (inclusion) remained within 3 days. The treatment of the index infarction in both groups was comparable. Of the invasive patients 53% received thrombolysis, compared to 45% of the conservative, the difference being non-significant. The time from onset of symptoms to the start of fibrinolytic therapy was also comparable in both groups. Cardiac catheterization was performed in 99% of patients in the invasive strategy group (1 patient died before the assigned catheterization). Of these patients, 73% received a percutaneous intervention, 11% underwent coronary-artery bypass surgery because of high risk anatomy and 16% did not receive a revascularization procedure at all. Most of these patients had non-significant coronary artery disease. In some patients the culprit vessel was too small for PCI. Medical therapy at discharge was similar in both randomized groups, except for the use of clopidogrel, which drug was off course obligatory in patients with a stent.

**Table 1:** Baseline characteristics of the patients

	Viable		Non-viable	
	Invasive	Conservative		P- value*
Characteristic	(n = 106)	(n = 110)	(n = 75)	
Male	75%	81%	66%	0.41
Age (yrs)	60	59	64	0.52
Clinical history (%)				
Angina	41%	44%	54%	0.68
Myocardial infarction	6%	4%	9%	0.53
Percutaneous coronary intervention	2%	3%	9%	1.0
Coronary- artery bypass grafting	0%	1%	1%	1.0
Risk factors (%)				
Dabetes mellitus	8%	13%	12%	0.26
Hypertension	27%	28%	30%	1.0
Hypercholesterolemia	19%	14%	18%	0.36
Current sigaret smoking	45%	40%	64%	0.49
Family history of CAD	33%	31%	20%	0.77
Medications at admission (%)				
Aspirin	17%	9%	14%	0.11
Beta- blocker	12%	12%	18%	1.0
Ca- inhibitor	9%	4%	8%	0.16
Statins	15%	6%	12%	0.02
ACE- inhibitor	8%	6%	15%	0.59
AT II antagonist	6%	6%	5%	1.0
Time from onset of symptoms To randomization-hr	73**± **32	69**± **25	78**± **31	0.53
Time from onset of symptoms To thrombolysis-minutes	184**± **155	200**± **147	190**± **112	0.65
Thrombolysis	53%	45%	47%	0.34
Anterior infarction	31%	33%	47%	0.88
Ejection Fraction (EF%)	52.7	54.7	53.5	0.32
Randomization to revascularization				
mean (days)	5.6			
median (days)	2			
Occluded IRA (%)	19.8			
Absence of collaterals (%)	63.2			
Protocol PCI (%)	73			
CABG (%)	11			
No revascularization (%)	16			

* Differences between the randomized groups are expressed with a P-value. Plus-minus values are means ± SD.

### Primary endpoint

**Figure 1:**
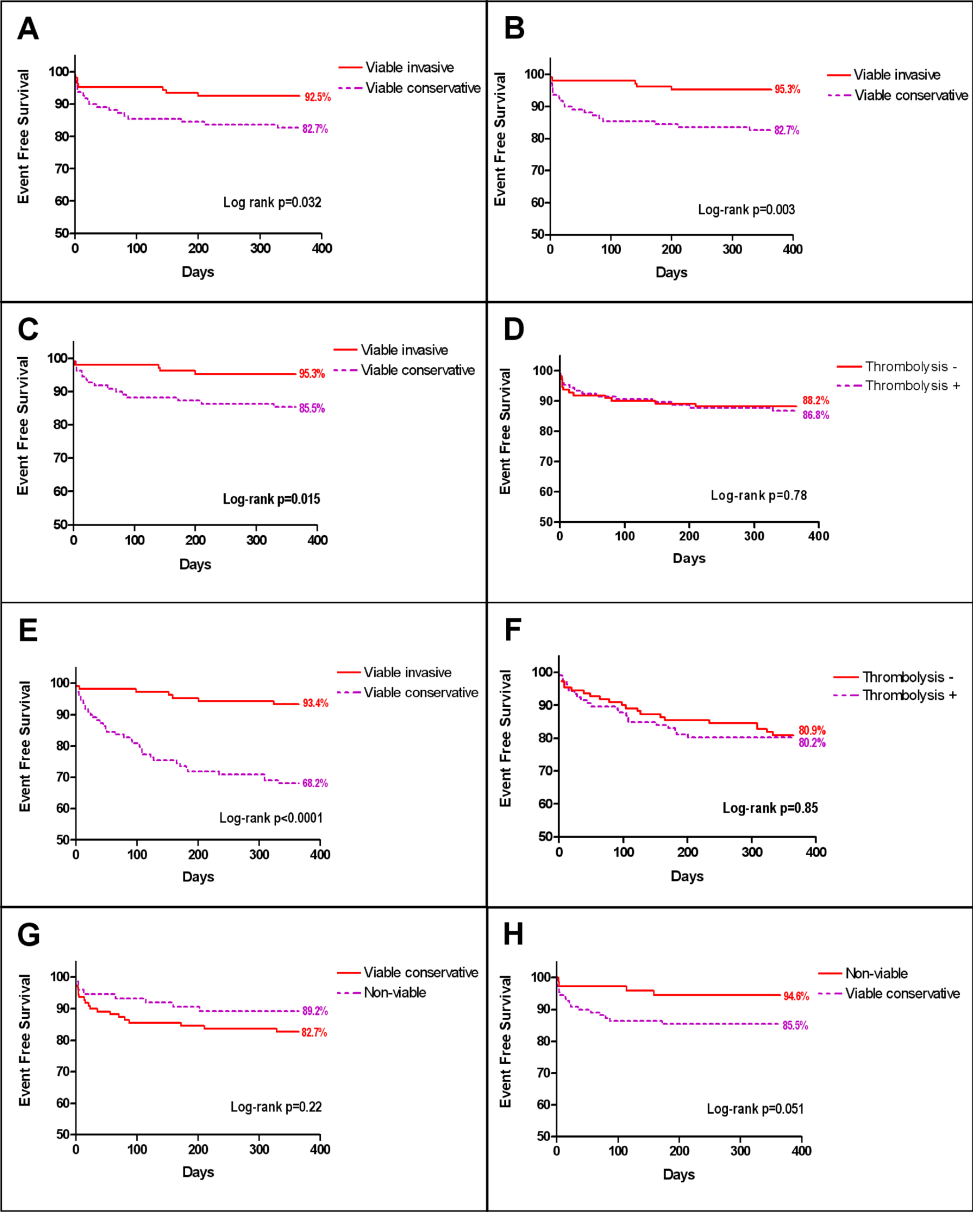
**Kaplan-Meier estimates of the cumulative rate of**; A. The composite primary end point of death from any cause, recurrent infarction and unstable angina within one year (intention to treat analysis). Viable invasive vs. viable conservative strategy. B. The composite primary end point of death from any cause, recurrent infarction and unstable angina within one year (per protocol analysis). Viable invasive vs. viable conservative strategy. C. The composite primary end point of death from any cause, recurrent infarction and unstable angina within one year (per protocol and adjusted for 2 days PCI delay). Viable invasive vs. viable conservative strategy. D. The composite primary end point of death from any cause, recurrent infarction and unstable angina within one year (intention to treat analysis). Patients who were treated with thrombolysis vs. patients who did not receive thrombolysis. E. The need for revascularization procedures after discharge and within one year. Viable invasive vs. viable conservative strategy. F. The need for revascularization procedures after discharge and within one year. Patients who were treated with thrombolysis vs. patients who did not receive thrombolysis. G. The primary composite endpoint (death, recurrent infarction and unstable angina) within one year. Non-viable patients (registry) vs. viable conservative patients (randomized). H. The cumulative rate of ischemic events (recurrent infarction and unstable angina) within one year. Non-viable patients (registry) vs. viable conservative patients (randomized).

The primary endpoint (death, recurrent MI and/or UA at one-year follow-up) occurred in 27 patients (8 in the invasive group and 19 in the conservative group). By intention-to-treat analysis the one-year event-free survival was 92.5% percent in the invasive strategy group and 82.7% in the conservative strategy group (Hazard ratio, 0.42; 95 percent confidence interval, 0.18 to 0.96; p = 0.04)(Figure 1A and Table 2). Mortality after one-year follow-up was 1.9% in the invasive group vs. 2.7% in the conservative group (p = 1.0). The cumulative risk of myocardial infarction within 12 months after randomization was comparable in both groups (1.9% in invasive vs. 1.8% in conservative group; p = 1.0). The risk of UA was significantly higher in the conservatively assigned group (12.7% vs. 3.7%; p = 0.02). As depicted in table 3 the effect of our intervention was more pronounced after adjustment for different variables (model c; Hazard ratio 0.31; CI 0.12 to 0.78; p = 0.01). The per-protocol analysis, which more accurately reflects the true influence of PCI on the occurrence of ischemic events, revealed a greater difference between the two randomized groups. The estimated one-year event-free survival by per-protocol analysis was 95.3 percent for the invasive strategy and 82.7 percent for the conservative strategy (Hazard ratio, 0.26; 95 percent confidence interval, 0.14 to 0.67; p = 0.003)(Figure 1B).

**Table 2:** Components of primary end points

	Invasive (n = 106)	Conservative (n = 110)	p-value*
Composite	8 (7.5%)	19 (17.3%)	0.04
Mortality	2 (1.9%)	3 (2.7%)	1.0
Acute MI	2 (1.9%)	2 (1.8%)	1.0
Unstable Angina	4 (3.7%)	19 (12.7%)	0.02

* P-values calculated with Fisher-exact test.

**Table 3:** Hazard ratios for composite primary end point (crude vs. adjusted models)

	Hazard Ratio (95% CI)	p- value*
Crude	0.42 (0.18 - 0.96)	0.04
Model a	0.43 (0.19 - 0.99)	0.05
Model b	0.33 (0.13 - 0.80)	0.02
Model c	0.31 (0.12 - 0.78)	0.01

* P-values calculated with cox proportional hazard regression analysis.

Model a; adjusted for DM, hypertension, hypercholesterolemia, current smoking, family history of CAD, Model b; adjusted for clinical history (angina, myocardial infarction, PCI or CABG) and medication use at baseline (aspirin, beta-blokker, Ca-inhibitor, statins, ACE-I and AT II antagonist), Model c; adjusted for all covariates used in model a and b

The Kaplan-Meier curves of the adjusted per-protocol analysis revealed an estimated one-year event free survival of 95.3 percent in the invasive group and 85.5 percent in the conservative group (Hazard ratio, 0.31; 95 percent confidence interval, 0.15 to 0.81; p = 0.015)(Figure 1C).

There was no difference in event-rate between patients who were treated with thrombolysis and patients who did not receive reperfusion therapy. Primary endpoint events were similar in these two groups (86.8% vs 88.2%; p = 0.78) (Figure 1D).

### Secondary endpoint

Patients in the invasive group had a significantly lower anginal class (CCS) (p = 0.021)(Table 4). After discharge there was a highly significant difference between the randomized groups in the need for revascularization procedures. In the invasive group only 6.6% (7/106) of patients underwent a new revascularization procedure compared to 31.8% (35/110) in the conservative group (p < 0.001)(Figure 1E). All but one procedures in the invasive group were primary end point driven. One revascularization occurred after proven ischemia (exercise ECG). The reasons for revascularization in the conservative group are depicted in table 5.

**Table 4:** Anginal class (CCS) in the randomized groups

Angina	Invasive	Conservative	p- value*
(CCS)	(n = 106)	(n = 110)	
I	87 (82.1%)	71 (64.5%)	0.021
II	13 (12.3%)	22 (20%)	0.021
III	5 (4.7%)	11 (10%)	0.021
IV	1 (0.9%)	6 (5.5%)	0.021

* P-value was calculated with chi-square test, Canadian Cardiovascular Society (CCS) classification.

**Table 5:** Components of secondary endpoints and reasons for revascularization (conservative and nonviable group)*

	Conservative	Nonviable	p- value
	(n = 110)	(n = 75)	
Angina (CCS)			
I	71 (64.5%)	45 (60.0%)	0.54
II	22 (20%)	15 (20%)	0.54
III	11 (10%)	12 (16%)	0.54
IV	6 (5.5%)	3 (4.0%)	0.54
Total revascularizations	35 (31.8%)	25 (33.3%)	0.87
- Primary endpoint driven	9 (8.2%)	1 (1.3%)	0.05
- Abnormal predischarge X-ECG	5 (4.5%)	7 (9.3%)	0.23
- Stable angina with proven ischemia	10 (9.1%)	1 (1.3%)	0.03
- Miscellaneous or non-ischemic chest discomfort	11 (10.0%)	16 (21.3%)	0.02
PCI (before hospital discharge)	6 (5.5%)	7 (9.3%)	0.38
PCI (within one year)	20 (18.2%)	10 (13.3%)	0.42
CABG (before hospital discharge)	1 (1.0%)	2 (2.7%)	0.57
CABG (within one year)	8 (7.3%)	7 (9.3%)	0.78
TIMI flow culprit vessel (CAG)	2.16	2.45	0.41

* Differences between the treatment groups are expressed with a P-value. CAG = coronary angiography

There was no difference in revascularization procedures performed in patients treated with thrombolysis and in those who received no reperfusion therapy (80.2% vs. 80.9%; p = 0.82)(Figure 1F).

### Outcome in the non-viable registry group

No significant difference in the primary composite endpoint was found in the comparison between the non-viable and the viable-conservative group (10.8% vs 17.3%; p = 0.22)(Figure 1G). In the analysis of the components of the primary endpoint we found a non-significant higher mortality in the non-viable group (5.3% vs. 2.7%; p = 0.44). Ischemic event-rate (recurrent MI and unstable angina) in the non-viable group was much lower than in the viable-conservative group (4.5% vs 14.5%; p = 0.051)(Figure 1H). Patients without viability underwent as many revascularization procedures as patients with viability who were treated conservatively (27.3% vs. 24%; p = 0.73)(Table 5). No differences were seen in revascularizations indicated by an abnormal pre-discharge exercise test (4.5% vs. 9.3%, p = 0.23). The need for primary endpoint driven revascularizations in the non-viable group tended to be lower than in the viable-conservative viable group (1.3% vs. 8.2%, p = 0.051). In the latter group revascularization procedures were more often performed based on objective evidence of ischemia (17.3% vs. 2.7%, p = 0.002). No difference in anginal status (CCS) was observed (Table 5).

## Discussion

The VIAMI-trial is the first RCT investigating a viability-guided invasive approach in patients who were at least 48 hours stable after acute MI (not treated with primary PCI), demonstrating that only patients with viable tissue in the infarct-area showed benefit from an early in-hospital culprit vessel revascularization.

Viability was used as a sensitive marker of risk of recurrent ischemia and recurrent infarction, with the notion that recurrent ischemic events in the post-MI period in most instances is related to re-thrombosis in the infarct-related coronary artery, combined with residual viable tissue in the infarct-area.

Several studies evaluated early invasive intervention after thrombolysis [19-25]. The only trials studying the effect of a routine invasive strategy after fibrinolysis beyond the time window of expected myocardial salvage were the GRACIA-1 and OAT. The TRANSFER-AMI and NORDISTEMI trials investigated this only in part. In the GRACIA-1 trial a routine invasive strategy within 24 hours (mean 19.6 hours) of thrombolysis in the modern era of PCI was investigated, compared with ischemia-guided conservative approach [25]. During the index hospital stay, no significant difference in hard endpoints (death or reinfarction) between the conservative and invasive treated group was demonstrated. Only the revascularization procedures differed significantly. At 1 year follow-up, a significant reduction of ischemia-related readmission to hospital and revascularization was observed without significant differences in non-fatal reinfarction or death. Both open and occluded arteries were included. The OAT trial included only patients with occluded arteries 3 to 28 days after AMI. Therefore, the OAT trial is the only trial addressing the routine opening of an occluded artery beyond the time window of expected myocardial salvage. The 4 year cumulative primary event rate (death, reinfarction or heart failure) did not differ significantly (17.2% PCI group vs. 15.6% medical group), with a trend toward excess reinfarction (p = 0.08) [26]. The TRANSFER-AMI trial compared high risk AMI patients treated with fibrinolysis and early PCI (< 6 hours) or fibrinolysis and standard-treatment. Overall, 88,7% of the patients in the standard group underwent coronary angiography after a mean of 32.5 hours. Urgent or rescue catheterization was performed within 12 hours after fibrinolysis in 34.9% of patients. After 6 months, no significant differences in incidence of death or reinfarction were demonstrated between the routine early PCI and standard treatment (p = 0.36) [20]. The same strategy was investigated in the NORDISTEMI trial. The standard treatment group underwent PCI 3 days (median) after AMI. Overall, in 95% of this group angiography was performed at a mean of 5.5 days. Urgent or rescue procedures within 12 hours were performed in 33%. No significant difference in primary endpoint was found after 1 year follow up (composite death, reinfarction, stroke, or new myocardial ischemia). Only composite of death, reinfarction and stroke differed significantly (p = 0.01) [24]. Comparing these trials with the VIAMI-trial is difficult, since we included only stable patients after AMI, representing a selected group of more or less low to intermediate risk. In the VIAMI trial no urgent or rescue PCI was performed as these patients were not included in the trial. In the conservative, non-invasive group of the VIAMI trial routine angiography was not recommended. Angiography was only performed in case of recurrent ischemia or proven ischemia with stress-testing. Overall, in the conservative group 41% (45/110) of patients underwent coronary angiography.

With regard to the recommended treatment post-thrombolysis, the AHA/ACC and ESC guidelines differ diminutively. The AHA/ACC guidelines recommend an early pharmacoinvasive strategy after thrombolysis in high risk patients (Class IIa, level B). In non-high risk patients such a strategy could be considered, especially if ischemic symptoms persist and failure to reperfuse is suspected (Class IIb, level C). The ESC guidelines and ESC guidelines for PCI recommend a routine invasive strategy within 24 hours after successful thrombolysis if available (Class IIa, level A). In stable patients who did not receive reperfusion therapy, angiography could be considered before discharge (Class IIb, level C) [1,2,27]. However, only the GRACIA-1 trial investigated a routine invasive strategy beyond the time window of expected myocardial salvage and demonstrated a reduction in endpoints (death, re-infarction and ischemia driven revascularization) after 12 months. In our opinion, insufficient for the generalized statement that routine intervention is warranted in patients after successful thrombolysis as recommended by the ESC guidelines.

As an unexpected finding, patients without viability underwent as many revascularization procedures as patients with viability who were randomized to the conservative strategy. Although patients without viability had a low recurrence-rate of acute coronary syndrome, they had similar reported CCS-class of angina compared to the viable-conservative patients. Despite a lower rate of spontaneous ischemic events and less proven ischemia during stress-testing, treating physicians decided to refer patients for an invasive procedure quite as often in the non-viable patient group. The explanation for this remains speculative.

The results of the VIAMI trial suggest that routine angioplasty in the early post-MI period in stable patients (whether or not treated with thrombolysis) is not mandatory. Especially in patients were the transport time for primary PCI is largely exceeding 90 minutes as recommended by the AHA/ACC guidelines, thrombolysis could be an interesting alternative with the VIAMI-trial approach. Also in patients presenting too late for reperfusion therapy (> 12 hours). Early viability-testing with low-dose dobutamine echocardiography provides us with a tool to identify high-risk patients who will have clinical benefit from a revascularization procedure later on.

### Limitations

The applicability of LDDE in daily practice is somewhat limited because about 10-15% of patients have poor acoustic windows. In our study this problem could partially be overcome with selective use of ultrasound contrast agents.

The pre-specified number of patients was not achieved. This was mainly caused by a slowing down of inclusion-rate in the second half of the study period due to the fast introduction of widely available primary PCI in the Netherlands. Although the primary event-rate in the viable-conservative group was lower than expected (17.3%) the relative risk-reduction was higher than expected (56.6%). This indicates an improvement over time in the pharmacological post-MI treatment (statins, clopidogrel, ACE-inhibitors). Also, it implicates further improvement in the efficacy and safety-profile of revascularization procedures. During the inclusion-period of the VIAMI-trial treatment with clopidogrel was not standard care in patients without stents. Standard treatment with clopidogrel according to current guidelines (CLARITY-TIMI 28 trial [28]) could have made the differences less pronounced.

The data from the non-viable patient group should be interpreted with caution, because this was a non-randomized group.

Half of the randomized patients were not treated with thrombolysis, making it a heterogeneous group. The outcome in these two seemingly different patient groups, however, was exactly the same. This finding challenges the general idea that patients with ST-segment elevation MI not treated with thrombolysis have completely different residual culprit vessel pathology with different early and long term clinical outcome than patients who were treated with thrombolysis. The VIAMI-trial supports the concept that, ultimately, it is viability that determines prognosis.

## Conclusion

Patients who are stable for 48 hours after acute myocardial infarction with viability in the infarct-area significantly benefit from an early invasive strategy. Importantly, patients who were not treated with thrombolysis showed the same benefit as patients who received thrombolytic therapy. An invasive approach in patients with viability results in a clear reduction in ischemic events and a long-term uneventful clinical course. The risk of recurrent ischemia is low in patients without viability.

## Competing interests

The VIAMI-trial was supported by The Netherlands Heart Foundation [2000B026 to GV], and The Interuniversity Cardiology Institute of the Netherlands (ICIN)[to GV and RvL]. GV received research grants from Eli Lilly, Boehringer Ingelheim Pharmaceuticals, and Bristol-Myers Squibb.

## Authors' contributions

RvL contributed to acquisition of data, analysis and interpretation of data, drafting the manuscript, and statistical analysis. GV contributed to this work in conception and design, interpretation of data, drafting of the manuscript, and supervision.

LB contributed to critical revision, and technical support. OK, JB, and AvR contributed to conception, design, and supervision. JT contributed to conception and design, statistical analysis, and supervision. All authors read and approved the final manuscript.

## References

[B1] Van de WF Bax J Betriu A Blomstrom-Lundqvist C Crea F Falk V Management of acute myocardial infarction in patients presenting with persistent ST-segment elevation: the Task Force on the Management of ST-Segment Elevation Acute Myocardial Infarction of the European Society of Cardiology Eur Heart J 2008 29 2909 2945 19004841 10.1093/eurheartj/ehn416

[B2] Kushner FG Hand M Smith SC Jr King SB III Anderson JL Antman EM 2009 focused updates: ACC/AHA guidelines for the management of patients with ST-elevation myocardial infarction (updating the 2004 guideline and 2007 focused update) and ACC/AHA/SCAI guidelines on percutaneous coronary intervention (updating the 2005 guideline and 2007 focused update) a report of the American College of Cardiology Foundation/American Heart Association Task Force on Practice Guidelines J Am Coll Cardiol 2009 54 2205 2241 10.1016/j.jacc.2009.10.015 19942100

[B3] Gunnar RM Passamani ER Bourdillon PD Pitt B Dixon DW Rapaport E Guidelines for the early management of patients with acute myocardial infarction. A report of the American College of Cardiology/American Heart Association Task Force on Assessment of Diagnostic and Therapeutic Cardiovascular Procedures (Subcommittee to Develop Guidelines for the Early Management of Patients with Acute Myocardial Infarction) J Am Coll Cardiol 1990 16 249 292 10.1016/0735-1097(90)90575-A 2197309

[B4] Eagle KA Nallamothu BK Mehta RH Granger CB Steg PG Van de WF Trends in acute reperfusion therapy for ST-segment elevation myocardial infarction from 1999 to 2006: we are getting better but we have got a long way to go Eur Heart J 2008 29 609 617 10.1093/eurheartj/ehn069 18310671

[B5] Veen G Meyer A Verheugt FW Werter CJ de Swart H Lie KI Culprit lesion morphology and stenosis severity in the prediction of reocclusion after coronary thrombolysis: angiographic results of the APRICOT study. Antithrombotics in the Prevention of Reocclusion in Coronary Thrombolysis J Am Coll Cardiol 1993 22 1755 1762 10.1016/0735-1097(93)90754-O 8245325

[B6] Basu S Senior R Raval U Lahiri A Superiority of nitrate-enhanced 201Tl over conventional redistribution 201Tl imaging for prognostic evaluation after myocardial infarction and thrombolysis [see comments] Circulation 1997 96 2932 2937 9386159 10.1161/01.cir.96.9.2932

[B7] Bigi R Desideri A Bax JJ Galati A Coletta C Fiorentini C Prognostic interaction between viability and residual myocardial ischemia by dobutamine stress echocardiography in patients with acute myocardial infarction and mildly impaired left ventricular function Am J Cardiol 2001 87 283 288 10.1016/S0002-9149(00)01359-X 11165961

[B8] Huitink JM Visser FC Bax JJ van Lingen A Groenveld AB Teule GJ Predictive value of planar 18F-fluorodeoxyglucose imaging for cardiac events in patients after acute myocardial infarction Am J Cardiol 1998 81 1072 1077 10.1016/S0002-9149(98)00143-X 9605044

[B9] Lee KS Marwick TH Cook SA Go RT Fix JS James KB Prognosis of patients with left ventricular dysfunction, with and without viable myocardium after myocardial infarction. Relative efficacy of medical therapy and revascularization Circulation 1994 90 2687 2694 7994809 10.1161/01.cir.90.6.2687

[B10] Nijland F Kamp O Verhorst PM de Voogt WG Visser CA In-hospital and long-term prognostic value of viable myocardium detected by dobutamine echocardiography early after acute myocardial infarction and its relation to indicators of left ventricular systolic dysfunction Am J Cardiol 2001 88 949 955 10.1016/S0002-9149(01)01968-3 11703987

[B11] Previtali M Fetiveau R Lanzarini L Cavalotti C Klersy C Prognostic value of myocardial viability and ischemia detected by dobutamine stress echocardiography early after acute myocardial infarction treated with thrombolysis J Am Coll Cardiol 1998 32 380 386 10.1016/S0735-1097(98)00243-5 9708464

[B12] Salustri A Ciavatti M Seccareccia F Palamara A Prediction of cardiac events after uncomplicated acute myocardial infarction by clinical variables and dobutamine stress test J Am Coll Cardiol 1999 34 435 440 10.1016/S0735-1097(99)00232-6 10440156

[B13] Sicari R Picano E Landi P Pingitore A Bigi R Coletta C Prognostic value of dobutamine-atropine stress echocardiography early after acute myocardial infarction. Echo Dobutamine International Cooperative (EDIC) Study J Am Coll Cardiol 1997 29 254 260 10.1016/S0735-1097(96)00484-6 9014975

[B14] Iskander S Iskandrian AE Prognostic utility of myocardial viability assessment Am J Cardiol 1999 83 696 702 A7 10.1016/S0002-9149(98)00973-4 10080421

[B15] van Loon RB Veen G Kamp O Bronzwaer JGF Visser CA Visser FC Early and long-term outcome of elective stenting of the infarct-related artery in patients with viability in the infarct-area: Rationale and design of the Viability-guided Angioplasty after acute Myocardial Infarction-trial (The VIAMI-trial) Current Controlled Trials in Cardiovascular Medicine 2004 5 10.1186/1468-6708-5-11 PMC534804 15538946

[B16] Bax JJ Wijns W Cornel JH Visser FC Boersma E Fioretti PM Accuracy of currently available techniques for prediction of functional recovery after revascularization in patients with left ventricular dysfunction due to chronic coronary artery disease: Comparison of pooled data Journal of the American College of Cardiology 1997 30 1451 1460 10.1016/S0735-1097(97)00352-5 9362401

[B17] Schiller NB Shah PM Crawford M DeMaria A Devereux R Feigenbaum H Recommendations for quantitation of the left ventricle by two-dimensional echocardiography. American Society of Echocardiography Committee on Standards, Subcommittee on Quantitation of Two-Dimensional Echocardiograms J Am Soc Echocardiogr 1989 2 358 367 2698218 10.1016/s0894-7317(89)80014-8

[B18] Platelet glycoprotein IIb/IIIa receptor blockade and low-dose heparin during percutaneous coronary revascularization. The EPILOG Investigators N Engl J Med 1997 336 1689 1696 9182212 10.1056/NEJM199706123362401

[B19] Armstrong PW A comparison of pharmacologic therapy with/without timely coronary intervention vs. primary percutaneous intervention early after ST-elevation myocardial infarction: the WEST (Which Early ST-elevation myocardial infarction Therapy) study Eur Heart J 2006 27 1530 1538 10.1093/eurheartj/ehl088 16757491

[B20] Cantor WJ Fitchett D Borgundvaag B Ducas J Heffernan M Cohen EA Routine early angioplasty after fibrinolysis for acute myocardial infarction N Engl J Med 2009 360 2705 2718 10.1056/NEJMoa0808276 19553646

[B21] Di Mario C Dudek D Piscione F Mielecki W Savonitto S Murena E Immediate angioplasty versus standard therapy with rescue angioplasty after thrombolysis in the Combined Abciximab REteplase Stent Study in Acute Myocardial Infarction (CARESS-in-AMI): an open, prospective, randomised, multicentre trial Lancet 2008 371 559 568 10.1016/S0140-6736(08)60268-8 18280326

[B22] Le May MR Wells GA Labinaz M Davies RF Turek M Leddy D Combined angioplasty and pharmacological intervention versus thrombolysis alone in acute myocardial infarction (CAPITAL AMI study) J Am Coll Cardiol 2005 46 417 424 10.1016/j.jacc.2005.04.042 16053952

[B23] Scheller B Hennen B Hammer B Walle J Hofer C Hilpert V Beneficial effects of immediate stenting after thrombolysis in acute myocardial infarction J Am Coll Cardiol 2003 42 634 641 10.1016/S0735-1097(03)00763-0 12932593

[B24] Bohmer E Hoffmann P Abdelnoor M Arnesen H Halvorsen S Efficacy and safety of immediate angioplasty versus ischemia-guided management after thrombolysis in acute myocardial infarction in areas with very long transfer distances results of the NORDISTEMI (NORwegian study on DIstrict treatment of ST-elevation myocardial infarction) J Am Coll Cardiol 2010 55 102 110 10.1016/j.jacc.2009.08.007 19747792

[B25] Fernandez-Aviles F Alonso JJ Castro-Beiras A Vazquez N Blanco J Alonso-Briales J Routine invasive strategy within 24 hours of thrombolysis versus ischaemia-guided conservative approach for acute myocardial infarction with ST-segment elevation (GRACIA-1): a randomised controlled trial Lancet 2004 364 1045 1053 10.1016/S0140-6736(04)17059-1 15380963

[B26] Hochman JS Lamas GA Buller CE Dzavik V Reynolds HR Abramsky SJ Coronary intervention for persistent occlusion after myocardial infarction N Engl J Med 2006 355 2395 2407 10.1056/NEJMoa066139 17105759 PMC1995554

[B27] Silber S Albertsson P Aviles FF Camici PG Colombo A Hamm C Guidelines for percutaneous coronary interventions. The Task Force for Percutaneous Coronary Interventions of the European Society of Cardiology Eur Heart J 2005 26 804 847 15769784 10.1093/eurheartj/ehi138

[B28] Sabatine MS Cannon CP Gibson CM Lopez-Sendon JL Montalescot G Theroux P Addition of clopidogrel to aspirin and fibrinolytic therapy for myocardial infarction with ST-segment elevation N Engl J Med 2005 352 1179 1189 10.1056/NEJMoa050522 15758000

